# Effects of Black Garlic Addition and Cooking Duration on Nitrosamine Levels and Quality Attributes of Sucuk

**DOI:** 10.3390/foods14234055

**Published:** 2025-11-26

**Authors:** Mükerrem Kaya, Asena Karayiğit, Zeynep Feyza Yılmaz Oral, Kübra Öztürk, Halenur Fencioğlu Çakır, Yağmur Akyol, Bilge Ulutaş, Selen Sallan, Güzin Kaban

**Affiliations:** 1 Department of Food Engineering, Faculty of Agriculture, Atatürk University, Erzurum TR-25240, Türkiye; asenakaryigit1997@gmail.com (A.K.); zeynep.yilmaz@atauni.edu.tr (Z.F.Y.O.); halenur.fencioglu@hotmail.com (H.F.Ç.); ygmr969@gmail.com (Y.A.); gkaban@atauni.edu.tr (G.K.); 2 NAMET R&D Center, Kocaeli TR-41420, Türkiye; ozturkkubra1516@gmail.com; 3 Quality Coordinatorship, Rectorate, Artvin Çoruh University, Artvin TR-08010, Türkiye; bilgeulutas@artvin.edu.tr; 4 Department of Food Processing, Bandırma Vocational School, Bandırma Onyedi Eylül University, Balıkesir TR-10200, Türkiye; ssallan@bandirma.edu.tr

**Keywords:** black garlic, fermented sausage, garlic, nitrosamine, NDMA, NPIP, sucuk

## Abstract

Sucuk, a type of dry fermented sausage, is generally consumed cooked, unlike many fermented sausage types. Therefore, alternative applications to prevent nitrosamine formation in fermented sausages such as sucuk are of great importance. This study aimed to determine the effects of black garlic (BG) and cooking time (0, 1, or 3 min at 180 °C) in sucuk. For this purpose, four different sucuk batters were prepared: FG1 (1% fresh garlic-control), BG1 (1% BG), BG2 (2% BG), and BG3 (3% BG). After ripening, the samples were cooked for different durations and subjected to nitrosamine analysis. Additionally, the study investigated the effect of BG on the qualitative properties of the ripened samples (uncooked). The results showed that the use of BG in sucuk led to a decline in lactic acid bacteria and *Micrococcus*/*Staphylococcus* counts (*p* < 0.05). The pH and residual nitrite levels in BG2 and BG3 were lower than those in FG1 and BG1 (*p* < 0.05). The TBARS value increased with increasing usage rate of BG (*p* < 0.05). BG2 and BG3 increased N-Nitrosodimethylamine (NDMA) compared to FG1 and BG1 (*p* < 0.05). Garlic treatment had no significant effect on the N-Nitrosodiethylamine (NDEA) (*p* > 0.05). As the cooking time increased, the NDMA and NDEA content increased (*p* < 0.05). The interaction between treatment and cooking time affected only N-Nitrosopiperidine (NPIP), and at the 3 min cooking time, BG3 exhibited a higher NPIP content than FG1, BG1, and BG2 (*p* < 0.05). As a result, BG2 and BG3 had a negative effect on some quality properties of sucuk, while FG1 and BG1 generally showed similar results. Additionally, BG2 and BG3 showed similar increases in NDMA content. Nitrosamine content increased with increasing cooking time, but BG3 was more effective in NPIP formation than BG2 after 3 min of cooking.

## 1. Introduction

Sucuk, a type of dry fermented sausage, is made without smoking or heat treatment [1]. Nitrate and/or nitrite are used as curing agents in this product, depending on the initial fermentation temperature. According to the Turkish Food Codex Regulation on Food Additives [2], the nitrite usage level was 150 mg/kg. The usage level of nitrate in sucuk production is also 150 mg/kg. Nowadays, nitrite is preferred as a curing agent in sucuk production since rapid ripening is generally performed [3]. Nitrite is a curing agent with significant effects on antimicrobial activity, retardation of lipid oxidation, cured flavor, and color development [4]. As well as these functions, it significantly contributes to nitrosamine formation. The reaction between secondary amines and a nitrosating agent produces nitrosamines [5]. Nitrite, which is not a nitrosating agent on its own, can be converted to nitrous acid (HNO_2_) in acidic environments, leading to the formation of reactive intermediates such as dinitrogen trioxide (N_2_O_3_) and nitrogen oxides (NO and NO_2_) [4,6]. N-nitrosodimethylamine (NDMA) and N-nitrosodiethylamine (NDEA) as probable carcinogens and N-nitrosodibutylamine (NDBA), N-nitrosopiperidine (NPIP), and N-nitrosopyrrolidine (NPYR) as possible carcinogens were classified by the International Agency for Research on Cancer [7]. Fermentation and drying involved in the manufacturing of dry fermented sausage, such as sucuk, also contribute to the formation of its precursors and the conditions necessary for nitrosamine formation. Protein and lipid degradation products during the ripening can be a good source for the formation of nitrosamine [5]. Biogenic amines can transform into secondary amines by deamination and cyclization and play a role as precursors of N-nitrosamines [8]. In addition to piperidine, which is supplied through the use of spices like pepper and is a major source of NPIP formation, cadaverine is thought to be a key precursor of NPIP in meat products. Lysine can also be a precursor for NPIP, and this amino acid can be converted into cadaverine by microorganisms that have decarboxylase activity [5,9]. Apart from these, during pyrolysis, lysine could undergo carbonyl-assisted decarboxylative deamination when sugars are included. This produces the Maillard product pent-4-en-1-amine, which can then cyclize into piperidine. Therefore, NPIP can arise when a mixture of lysine, glucose, and nitrite is heated [5]. It may consist of dimethylamine, lysine, methionine, choline, lecithin, carnitine, and glycine, which is the precursor of NDMA, another nitrosamine commonly detected in fermented sausages [8]. Alanine plays an important role in the formation of NDEA [5]. Additionally, the conversion of spermidine, putrescine, and proline to NPYR could be possible [5,10]. Adding nitrite to dry samples and heating at 170 °C transformed pyrrolidine ring-containing compounds to NPYR [5]. On the other hand, nitrosamine formation is also influenced by many factors, including residual nitrite level, a_w_, pH, spices (especially black pepper), starter culture, cooking temperature and time, and the presence of nitrosation catalysts/inhibitors as well as ingoing nitrite level [5,10,11,12,13,14,15].

Although sucuk is a dry fermented sausage type, it is usually cooked before consumption with dry heat treatments. A study investigating the effect of cooking on nitrosamine formation in sucuk has revealed that cooking is the most important risk factor in this product, and it has been reported that nitrosamine levels increase as cooking intensity increases [12]. Due to the harmful effects of nitrosamines, studies on preventing the nitrosamine formation in these products are of great importance [5,6,7,8,9,10,11,12,13,15]. Antioxidants such as ascorbic acid, phenolic compounds, sulfur compounds, plant phenols, and various inhibitory substances are reported to have an important role in inhibiting the nitrosation reaction [15,16,17,18,19,20,21,22]. Antioxidants reduce nitrosamine formation by promoting the reduction in nitrite and nitrous acid, and by inhibiting the oxidation of nitric oxide. These compounds also help to reduce nitrosamine formation by blocking the reactions that cause the formation of free radicals [23,24].

Garlic (*Allium sativum* L.) has long been known for its therapeutic benefits [25]. Many studies have reported on the beneficial effects of garlic for human health, as it is a plant that includes polyphenols, sulfur compounds, and selenium, as well as significant antioxidant capacity and antibacterial capabilities due to the phenolic compounds, saponins, and proteins it contains [26,27]. On the other hand, black garlic, obtained by fermenting white garlic at high relative humidity and temperatures, has attracted attention because of its high antioxidant content [26]. Changes in the physicochemical properties of black garlic during its production are due to modifications or interactions that occur in carbohydrates, free amino acids, polyphenols, volatile sulfur compounds, and other antioxidant compounds. These changes significantly increase the bioactivity of black garlic [28]. The rich sulfur compound in fermented garlic (black garlic) has the potential to have an important inhibitory effect on the formation of nitrosamine [29,30]. However, in a study on heat-treated sucuk, a semi-dry fermented sausage produced by a process of fermentation, heat treatment, and drying, it was found that black garlic inhibited the formation of NDMA and NDEA, while increasing the level of NPIP [31]. However, there are no studies in the literature on the influence of garlic (unprocessed, fresh) or black garlic on the formation of nitrosamines in sucuk, a traditional dry fermented sausage produced without heat treatment.

The study aimed to determine the effects of fresh garlic (1%) and different levels of black garlic (1%, 2%, and 3%) on the quality characteristics of sucuk. For this purpose, ripened sucuk samples (final product, uncooked) were subjected to physicochemical and microbiological analyses. The study also aimed to evaluate the effect of cooking time on nitrosamine formation in sucuk produced with fresh garlic or different levels of black garlic. The ripened sucuk samples were subjected to nitrosamine analysis after cooking for different times (0, 1, and 3 min).

## 2. Materials and Methods

### 2.1. Material

In the study, beef meat and beef fat were used from beef carcasses purchased from a local butcher in Erzurum. Beef meat was chopped into small pieces after the removal of excess fat, vacuumed, and then kept in storage (−18 °C) until production. The sucuk production was conducted at three different times using beef meat and beef fat from 3 different beef carcasses. Fresh garlic (Taşköprü garlic named as Taşköprü sarımsağı) (pH: 6.33; aw: 0.973) and black garlic (pH: 4.48; a_w_: 0.907) were purchased from the firm (Orhan Reis Tarım Ürünleri A.Ş., Taşköprü, Kastamonu, Türkiye). Autochthonous *Latilactobacillus sakei* S15 (10^7^ cfu/g) and *Staphlococcus xylosus* GM97 (10^6^ cfu/g) strains [32] were added as starter cultures.

### 2.2. Sucuk Production

In the production, 7 g red pepper, 9 g cumin, 20 g salt, 5 g black pepper, 2.5 g allspice, 0.15 g sodium nitrite, and 4 g sucrose were included in the formulation for a 1 kg meat + fat mixture (200 g meat fat + 800 g lean meat) [12]. Four formulations were prepared: FG1: 1% fresh garlic; BG1: 1% black garlic; BG2: 2% black garlic; and BG3: 3% black garlic. Three independent manufacturing processes were carried out for each treatment (three batters per treatment). Thus, a total of 12 sucuk batches (4 treatment × 3 replications) were prepared.

A laboratory-type cutter (MTK 662, Mado, Dornhan, Schwarzwald, Germany) was utilized to make the sucuk batters, which were then stuffed into collagen casings (38 mm, Naturin GmbH & Co., Weinheim, Germany) in batches of 200 g each. The prepared sucuk samples were taken to the automatic climate unit (Reich, Schechingen, Germany), where the temperature, air flow, and relative humidity can be controlled automatically. The ripening (fermentation and drying) program was as follows: temperature and relative humidity were 24 ± 1 °C at 90 ± 2% RH for the first day, 18 ± 1 °C at 90 ± 2% RH for the second day, 16 ± 1 °C at 86 ± 2% RH for 3rd, 4th, and 5th days; and 16 ± 1 °C at 84 ± 2% RH for the 6th and 7th days. Air flow, which was kept at 0.5 m/s for the first 3 days of ripening, progressively decelerated to 0.1 m/s over the following days.

### 2.3. Cooking Procedure for Sucuk

Before cooking, the samples were cut into 0.5 mm-thick slices. The samples were then cooked at 180 °C on a hotplate (Testo, Titisee-Neustadt, Germany) with a digital thermocouple. Cooking times of 1 min (0.5 min each surface) and 3 min (1.5 min per surface) were performed. The uncooked samples were evaluated as the control group. Before being analyzed, every sucuk sample was homogenized and frozen at −20 °C in a glass jar.

### 2.4. Analysis

TBARS, pH, a_w_, residual nitrite, and microbiological analyses were carried out on the final products. Nitrosamine analysis was undertaken on uncooked (0 min) as well as cooked samples (1 or 3 min).

### 2.5. Microbiological Analysis

In order to investigate the lactic acid bacteria count of the sucuk samples belonging to each treatment group, inoculation was carried out using the spreading technique on de Man Rogosa Sharpe (MRS) Agar (Merck, Darmstadt, Germany) plates and then incubated anaerobically at 30 °C (Anaerocoult A, Merck). After incubation, the number of lactic acid bacteria was determined by considering the catalase (-) colonies, and the results are given as log cfu/g. Mannitol Salt Phenol Red Agar (MSA, Merck) was used for determining the number of *Micrococcus*/*Staphylococcus* count by incubating at 30 °C for 48 h. For the purpose of counting Enterobacteriaceae, Petri plates with Violet Red Bile Dextrose (VRBD, Merck) agar were utilized. Petri plates were incubated for two days at 30 °C in anaerobic conditions (Anaerocoult A) [1].

### 2.6. Physicochemical Analysis

#### 2.6.1. pH and Water Activity (aw) Determination

The sucuk sample (10 g) was added to 100 mL of pure water, and the mixture was homogenized using an ultra-turrax (IKA Werk T25, Staufen im Breisgau, Germany). A pH meter (Mettler Toledo, Greifensee, Switzerland) was utilized to determine the pH value. A water activity device (Novasina AG CH-8853, Pfäffikon, Switzerland) was employed. Before the device was used, it was subjected to a calibration process with six different salt solutions at 25 °C.

#### 2.6.2. Thiobarbituric Acid Reactive Substances (TBARS) Analysis

The method specified by Kilic and Richards [33] was applied for TBARS analysis. Two g samples were homogenized with 12 mL trichloroacetic acid (TCA) solution prepared with 7.5% TCA, 0.1% ethylene diamine tetraacetic acid, and 0.1% propyl gallate. After filtering (Whatman 1) the homogenate, 3 mL of thiobarbituric acid solution (0.02 M) was added to it, and the samples were kept in a boiling water bath for 40 min. After cooling, the mixture was centrifuged for 5 min. The measurement was performed at 530 nm. For a standard curve, 1,1,3,3-tetraethoxypropane was used. The findings were given as mg MDA/kg.

#### 2.6.3. Residual Nitrite Analysis

The samples (10 g) were added to 50 mL of ultrapure hot water (50–60 °C) and mixed. Then, the mixture was transferred into a 200 mL volumetric flask and added to acetonitrite (50 mL). After cooling to room temperature, it was diluted with water to the mark and filtered through the fluted filter paper (MN 640 de, Macherey-Nagel, Düren, Germany). Afterward, the filtrates were passed through a 0.45 µm filter and placed in vials. The samples were analyzed through the HPLC/DAD (Agilent 1100, Santa Clara, CA, USA). Hamilton PRP-X100 column (5 µm, 150 × 4.6 mm, Santa Clara, CA, USA) was used in the system. The UV wavelength used for detection was 220 nm, the injection volume was 100 µL, and the flow rate was set at 2 mL min^−1^. The mobile phase was prepared as follows: First, 17 mL of lithium borate gluconate buffer solution and acetonitrile (125 mL) were added to 500 mL of water in a 1000 mL volumetric flask. The solution was diluted with water to the mark and mixed well, then the pH was adjusted to 6.5. For validation of analysis, various concentrations of 5–30 mg/L standard solutions containing nitrite were spiked into samples (five replicates). Mean recoveries and relative standard deviations (RSDs) ranged from 98.02% to 103.64% and from 1.34% to 2.75%, respectively. The limits of detection (LOD) and limit of quantification (LOQ) were determined by different concentrations (1–50 mg/L) of standard and using the following formula: LOD =3.3 × Sy/s and LOQ =10 × Sy/s (Sy: the standard deviation of the response of the curve, and s: the slope of the calibration curve). LOD and LOQ were determined as 1.49 mg/L and 4.52 mg/L, respectively. Intra-day and inter-day accuracy were calculated by adding a concentration of 10 mg/L standard to the sample, measuring five replicates, and repeating for three days. The relative standard deviations (RSDs) were satisfactory, and the intra-day repeatability was 1.32%, while the inter-day reproducibility was 2.12%. The coefficient of the regression line (R2) was 0.9998 (Figure S1). The results were expressed as mg/kg [34].

#### 2.6.4. Instrumental Color Measurement

The color values (L*, a*, and b*) of the samples that were cut into slices of 1 cm thickness were obtained using a colorimeter (Minolta Co., Osaka, Japan). It included an 8 mm aperture, a standard observed of 2°, and a *C D65 illuminant.

### 2.7. Nitrosamine Analysis

The method given by Wang et al. [15] was performed for nitrosamine analysis. A homogeneous sample (10 g) was transferred to the centrifuge tube, and homogenization was performed after adding the NaOH solution. After adding methanol to the homogenizer, it was centrifuged (11.963× *g* at 4 °C). After the concentrating process, filtration was made with glass microfiber (Whatman GF, Maidstone, UK), and a 20% NaCl solution was added and transferred to the ChemElut column (Agilent, Santa Clara, CA, USA). Then, using the Kuderna Danish apparatus, the diluent was concentrated to 1 mL following the addition of dichloromethane. The concentrate was evaporated using nitrogen at 40 °C. Nitrosamines were determined using GC/MS (Agilent 6890N/Agilent 5973, Santa Clara, CA, USA). In the system, carrier gas was helium, and the column was DB-5MS (30 m × 0.25 mm × 0.25 µm, Agilent, USA). The identification was carried out in SIM mode. The oven temperature was held at 50 °C for 2 min, then quickly raised to 100 °C by 3 °C/min and held for 5 min. After this process, the temperature was raised to 250 °C at a speed of 20 °C/min. Nitrosamine mix (EPA 521, Supelco, Bellefonte, PA, USA) was used in the identification, and according to the external standard method, the amounts of nitrosamine (NDMA, NDEA, NDPA, NPIP, NMEA, NPYR, and NDBA) were determined as µg/kg (Figure S2). The different concentrations of 5–20 mg/L standard solutions were spiked into samples (five replicates), and mean recoveries and relative standard deviations (RSDs) for NDMA, NDEA, NPYR, NPIP, NDBA, and NDPA were given in Table S1. Intra-day and inter-day accuracy were calculated by adding standards to the sample at three concentration levels, measuring five replicates, and repeating for three days (Table S1, Figure S2). The limit of detection (LOD) and limit of quantification (LOQ) were determined by different concentrations (0.5–20 µg/mL) of standard and using the following formula: LOD = 3.3 × Sy/s and LOQ = 10 × Sy/s (Sy: the standard deviation of the response of the curves: the slope of the calibration curve) (Table S1).

### 2.8. Statistical Analysis

The study was based on four treatments (control (1% fresh garlic-FG1), 1% black garlic-BG1, 2% black garlic-BG2, and 3% black garlic-BG3) with three replications (blocks). The trials were conducted according to a randomized complete block design plan. The cooking time (raw-uncooked, 1 min, and 3 min) was also considered as a factor for the nitrosamine analysis, and the trial was carried out according to a randomized complete block design plan in a 3 × 4 factorial design. Data was assessed by two-way analysis of variance using a general linear model. Garlic treatment (FG1, BG1, BG2, and BG3) was the main effect for microbiological analysis, pH, a_w_, TBARS, color (L*, a*, and b*), and residual nitrite. Garlic treatment and cooking time (for nitrosamine analysis) were regarded as fixed factors, and for all analyses, the replicates were performed as a random effect. The means of the significant parameters were compared with the Duncan multiple comparison test at *p* < 0.05. A SPSS 24 (SPSS Inc., Chicago, IL, USA) statistical program was used for all statistical analyses. Furthermore, the cluster heat map showing the differential profile of the nitrosamines in the samples (raw for 0 min or cooked for 1 or 3 min) made with different garlic treatments (FG1, BG1, BG2, or BG3) was obtained using a heat mapper [35].

## 3. Results and Discussion

### 3.1. Microbiological Results

**Figure 1:**
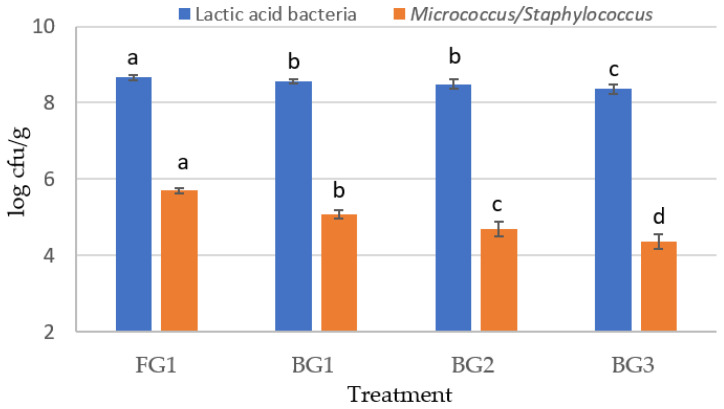
The effect of using garlic on lactic acid bacteria and *Micrococcus*/*Staphylococcus* counts of sucuk (FG1: 1% fresh garlic; BG1: 1% black garlic; BG2: 2% black garlic; and BG3: 3% black garlic). (a–c: Different lowercase letters indicate significant differences between treatments for lactic acid bacteria. a–d: Different lowercase letters indicate significant differences between treatments for *Micrococcus*/*Staphylococcus*).

The effect of using garlic on lactic acid bacteria and *Micrococcus*/*Staphylococcus* counts of sucuk is shown in Figure 1. The highest mean of lactic acid bacteria (LAB) was determined in the FG1 (control) group. While there was a significant difference in the BG1 and BG2 groups, the lowest mean LAB count was found in BG3 (Figure 1). It is thought that these results are due to the increase in antimicrobial activity with the increase in the use of BG [36]. However, there was a desirable level of LAB count even for the BG3 level. On the other hand, it was reported that BG2 and BG3 caused a decline in the number of LAB in heat-treated sucuk [31]. LAB are important microorganisms for fermentation in sucuk and similar fermented products. Acidification formed by these microorganisms is essential for both product safety and the development of some sensory features of the product. In the present study, *Latilactobacillus sakei* S15 was added to sucuk batters as a starter culture. According to the results, this strain showed a good adaptation to the sucuk environment at the initial fermentation temperature of 24 ± 1 °C and significantly maintained its survival in the final product. Previous studies have also reported that the LAB strains used as starter cultures have adapted well to the sucuk environment, and the number was around 10^8^ cfu/g [1,37,38].

The lowest mean *Micrococcus*/*Staphylococcus* count was observed in the control group (Figure 1). In the groups containing black garlic, the number decreased as the black garlic level increased, and the differences among the groups were significant (*p* < 0.05). *Micrococcus*/*Staphylococcus* significantly affected the rate and degree of acidification of fermented sausages during the fermentation stage. These microorganisms show little or no growth in the fermentation environment. Therefore, the strains utilized as starter cultures are added to fermented sausage batters at a minimum level of 10^6^ cfu/g [1]. In this study, the number of *Staphylococcus xylosus* added to the sucuk batter was at the level of 10^6^ cfu/g, and the starter culture decreased to 10^4^ cfu/g in the final product. However, in other studies in which this strain was used, *Micrococcus*/*Staphylococcus* count in the final product was found above 1 × 10^6^ cfu/g [37]. In the present study, the low number of *Micrococcus*/*Staphylococcus* in the final product indicates that the pH decreased rapidly at the initial fermentation temperature of 24 °C. Since these microorganisms are acid-sensitive, they are affected by the rapid pH decrease during fermentation [39]. In this study, it is assumed that the low *Micrococcus*/*Staphylococcus* counts detected in the black garlic groups are due to the low pH of the black garlic used (pH: 4.48). The higher content of fermentable sugars in black garlic compared to white garlic is believed to contribute to the rapid drop in pH during fermentation. Choi et al. [40] reported that the content of reducing sugars in black garlic was higher than that in fresh garlic. Yuan et al. [41] also determined a water-soluble sugar content of 187.79% in fresh garlic and 790.96% in black garlic. Furthermore, the antimicrobial activity of black garlic is thought to contribute to this result. Indeed, organic sulfur compounds, considered to be the key components of black garlic, have been reported to increase antimicrobial activity [36].

### 3.2. Physicochemical Properties

There was no significant difference between the FG1 (control) and BG1 samples for pH. A significant decrease in pH value was obtained when BG was used at a 2% or 3% level (*p* < 0.05). But no significant difference was observed between these groups in terms of pH (*p* > 0.05) (Table 1). In fermented products, lactic acid bacteria are used as starter cultures or spontaneously utilize the sugars from the environment to form lactic acid and, thus, decrease the pH [42]. In this study, pH decreased more in groups containing more than 1% BG. As seen from Figure 1, the number of lactic acid bacteria in these groups was found to be 0.5 logarithmic units lower compared to the control group with FG. The remarkable result here is that the number was found above 1 × 10^8^ cfu/g, as in the control group. Regarding a_w_, the lowest a_w_ mean was obtained in the FG1 group and was not statistically different from the a_w_ of the BG1 group (*p* > 0.05). The high pH values of the BG2 and BG3 groups are probably due to the low a_w_ value of black garlic.

The highest TBARS value was found in the BG3 group, while the lowest TBARS value was determined in the control group (FG1). Usage of BG3 significantly increased lipid oxidation in sucuk (*p* < 0.05) (Table 1). This result is thought to be likely due to the prooxidant effect of some compounds in BG [31]. Indeed, it is suggested that aldehydes, alcohols, and ketones formed during the production of BG are effective in some chemical reactions [43,44]. On the other hand, some phenolic compounds have been reported to exhibit prooxidant (electron acceptor) effects depending on environmental conditions [45]. Additionally, the prooxidant activity of fresh and black garlic extracts was emphasized as being dependent on concentration [46].

Nitrite is a multifunctional curing agent and plays a significant role in the formation of the characteristic color, the inhibition of oxidation, and the formation of cure flavor. Another feature of this additive is that it has antimicrobial activity [4]. In the study, no statistical difference was observed between the mean residual nitrite values of the control and BG1 group. On the other hand, 2% and 3% levels of BG decreased the amount of residual nitrite (*p* > 0.05) (Table 1). One of the most important factors affecting residual nitrite amount in cured meats is pH decrease during fermentation. As the pH decreases during fermentation, the residual nitrite level decreases significantly [4]. In this study, the fact that BG2 and BG3 gave lower nitrite levels than the other groups is thought to be related to the pH values of these groups.

### 3.3. Instrumental Color

**Figure 2:**
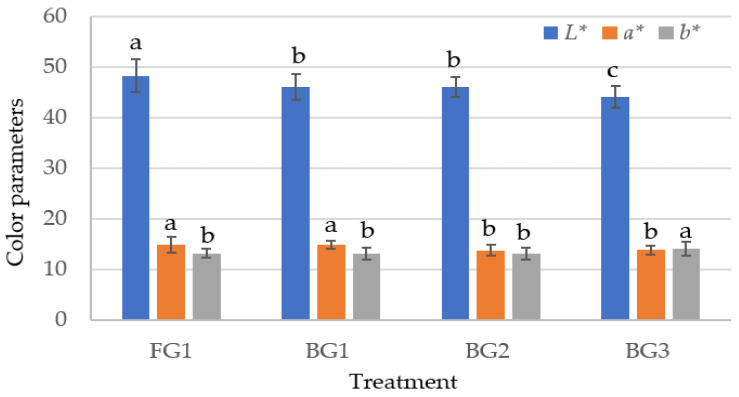
The effect of using garlic on the color parameters of sucuk (a–c: Different lowercase letters indicate significant differences between treatments for each color parameter).

L* value, which provides information about the brightness of the product, decreased significantly at BG3. The highest L* value was detected in the control group. In the BG1 and BG2 groups, L* value decreased compared to the control group, but no significant difference was detected between the mean of these two levels of BG (*p* > 0.05) (Figure 2). These differences are thought to be related to the a_w_ value of the product. As for a* value, which is used in the evaluation of red color intensity, no significant difference was found between the control group and 1% BG group. On the other hand, a* value decreased at 2% and 3% levels. a* value of sucuk influenced by black garlic usage is thought to be related to the Maillard reaction products during the black garlic production process [47]. The BG3 group gave the highest b* value among the sucuk groups (*p* < 0.05). No statistically significant difference was observed between the other groups (FG1, BG1, and BG2) (Figure 2).

### 3.4. Nitrosamines

NDMA is a nitrosamine widely detected in fermented sausages [11,48,49]. The overall effects of cooking time and garlic treatment on the nitrosamine levels of sucuk are shown in Table 2. The control group had the lowest NDMA value found in the study. There was no important difference observed between the mean value of the control and the BG1 group (*p* > 0.05) (Table 2). Higher means, however, were seen at the BG levels of 2% and 3%. Nevertheless, no significant statistical difference was obtained between the means of these levels (Table 2). These findings indicated that the 2% and 3% BG treatments exhibited a similar increase in the amount of NDMA in sucuk. However, in a study on heat-treated sucuk (pH: 5.1–5.2), a semi-dry fermented sausage, BG had no significant effect in samples that were not heat-treated again [31]. The higher NDMA levels in this research were most likely attributable to the lower pH (4.68–4.81) (Table 1) and longer ripening time. As seen in Table 2, the BG2 and BG3 groups had higher NDMA levels than the BG1 and FG1 groups. Although BG2 and BG3 had lower residual nitrite levels than the other groups, this difference is likely to be due to pH. These results indicate that in fermented sausages, in addition to pH, ripening conditions are also effective in nitrosamine formation. On the other hand, NDMA content rose with cooking time, but there was no interaction between cooking time and garlic treatment in terms of NDMA. However, in the study conducted by Akansel et al. [31], 3% BG level decreased NDMA content in samples cooked for 1 min and 3 min. The significant effect of cooking on NDMA content in sausage was also revealed in other studies [12,50,51].

As for NDEA, no significant difference between garlic treatment groups was found (*p* > 0.05). NDEA content in raw samples was found below the detectable limit (Table 2). Parallel results were also found in a study conducted on sucuk [50]. On the other hand, NDEA increased depending on cooking time in the present study. In research conducted on raw sausage, it was reported that frying increased the NDEA content [52]. Similar results were found by Rywotycki [53] and Lu et al. [54]. According to Akansel et al. [31], black garlic is more effective in preventing NDEA formation during cooking since it includes many antioxidant components.

**Figure 3:**
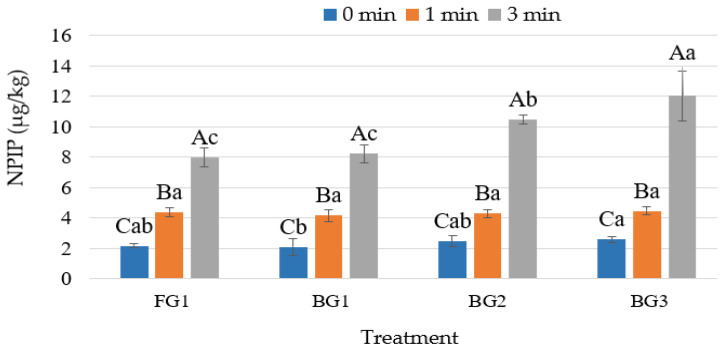
Effect of using garlic and cooking time interaction on N-Nitrosopiperidine (NPIP) content of sucuk (A–C: Different capital letters indicate significant differences between cooking time for garlic treatment, and a–c: Different small letters indicate significant differences between garlic treatment for cooking time).

Black pepper contains piperine and piperidine, which are key precursors of NPIP [5,55]. In contrast to NDEA and NDMA, greater mean NPIP values were detected in raw samples in the current investigation (Table 2). Precursor compounds generated as a result of the changes in amino acids, lipids, and proteins that occurred [23] during the seven-day ripening process of sucuk are assumed to be the cause of this finding. The NPIP level of sucuk increased with cooking time in all garlic groups, as shown in Figure 3. At 3 min of cooking, a further increase in NPIP content was noted in the BG2 and BG3 groups. This finding indicates that certain components of BG are more effective in forming NPIP during 3 min of cooking. The BG contains lysine, a precursor for NPIP [9], in greater amounts than FG [56]. Furthermore, it is thought that the higher glucose content of BG compared to FG [57] may cause an increase in NPIP content during cooking. Indeed, De Mey et al. [5] reported that the presence of glucose, nitrite, and lysine led to NPIP formation during heat treatment. Additionally, other studies have also demonstrated that the NPIP increased with an increase in cooking time or degree [12,31,58].

**Figure 4:**
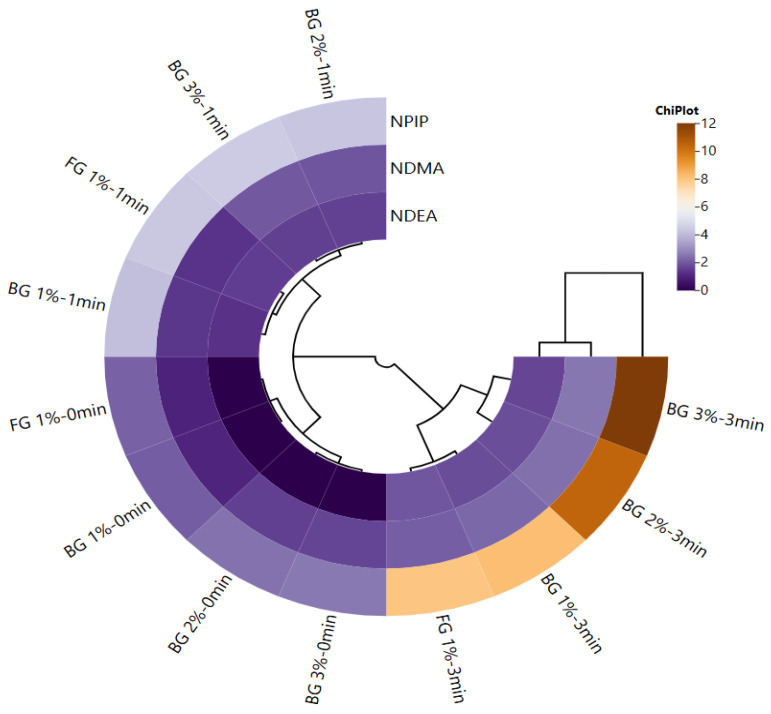
Cluster analysis of a heat map showing the relationship between garlic treatments (fresh garlic: FG, and black garlic: BG) and cooking time (0, 1, and 3 min) for nitrosamines.

The current study examined the general differences between samples cooked at three different times (0, 1, or 3 min) using a cluster analysis based on the level of nitrosamines in sucuk. Figure 4 showed that NDMA and NDEA contents were similar to each other, while NPIP showed a different content from these two nitrosamines. When the BG ratio and cooking time were taken into account, three clusters were formed. These clusters were divided into two subclusters: FG1 and BG1 were in the same cluster, and BG2 and BG3 were in the same cluster. It was determined that uncooked (0 min) samples had the lowest values in all groups. The extension of the cooking time increased NPIP content, and the highest values in all groups were observed in 3 min of cooking. On the other hand, 2% or 3% ratios of BG increased the NPIP content considerably. This result indicates the presence of precursors for NPIP formation, especially in BG.

## 4. Conclusions

The use of black garlic in sucuk affected the technologically important lactic acid bacteria and *Micrococcus*/*Staphylococcus* counts. However, lactic acid bacteria grew up to 8 log cfu/g and caused a sufficient pH decrease. The BG also contributed to the pH decrease. Low pH values, however, had a significant impact on the number of *Micrococcus*/*Staphylococcus*, and the BG3 treatment led to a reduction in the number of more than 1 log cfu/g compared to FG. Additionally, this amount of black garlic also increased lipid oxidation (TBARS value > 1 mg MDA/kg). Using BG at 1% yields similar nitrosamine levels and quality attributes to FG, while higher BG inclusions (≥2%) increased NDMA and NPIP, particularly with cooking. To limit nitrosamine content, BG should, therefore, not be used in quantities exceeding 1%. The BG2 and BG3 treatments, particularly after 3 min of cooking, lead to NPIP contents exceeding 10 ug/kg. To minimize the risk of nitrosamines, the product should ideally be consumed directly, without heat treatment. In addition, studies on the effects of spices (including black pepper) and antioxidants on nitrosamine formation in sucuk are considered necessary.

## Figures and Tables

**Table 1:** The overall effects of using garlic on pH, a_w_, TBARS, and residual nitrite levels of sucuk (mean ± standard deviation).

Treatment	pH	a_w_	TBARS (mg MDA/kg)	Residual Nitrite (mg/kg)
FG1	4.81 ± 0.03 ^a^	0.909 ± 0.003 ^a^	0.58 ± 0.08 ^d^	23.87 ± 2.92 ^a^
BG1	4.78 ± 0.05 ^a^	0.906 ± 0.003 ^ab^	0.73 ± 0.04 ^c^	25.27 ± 1.95 ^a^
BG2	4.70 ± 0.03 ^b^	0.902 ± 0.006 ^b^	0.88 ± 0.10 ^b^	22.00 ± 1.69 ^b^
BG3	4.68 ± 0.02 ^b^	0.902 ± 0.005 ^b^	1.27 ± 0.27 ^a^	22.13 ± 1.99 ^b^

FG1: 1% fresh garlic; BG1: 1% black garlic; BG2: 2% black garlic; and BG3: 3% black garlic; ^a–d^: Means marked with different letters in the same column are statistically different (*p* < 0.05).

**Table 2:** The overall effects of using garlic and cooking time on the nitrosamine levels (µg/kg) of sucuk (mean ± standard deviation).

Treatment	n	NDMA	NDEA	NPIP
Garlic (G)				
FG1	18	1.47 ± 0.54 ^b^	1.16 ± 0.88 ^a^	4.84 ± 2.49 ^c^
BG1	18	1.55 ± 0.62 ^b^	1.04 ± 0.79 ^a^	4.81 ± 2.67 ^c^
BG2	18	1.96 ± 0.45 ^a^	1.13 ± 0.87 ^a^	5.74 ± 3.54 ^b^
BG3	18	2.06 ± 0.55 ^a^	1.06 ± 0.80 ^a^	6.36 ± 4.30 ^a^
Cooking Time (CT)				
0 min	24	1.29 ± 0.39 ^c^	<LOD	2.32 ± 0.39 ^c^
1 min	24	1.66 ± 0.41 ^b^	1.49 ± 0.24 ^b^	4.32 ± 0.32 ^b^
3 min	24	2.33 ± 0.37 ^a^	1.80 ± 0.28 ^a^	9.67 ± 1.93 ^a^
G × CT		ns	ns	**

FG1: 1% fresh garlic; BG1: 1% black garlic; BG2: 2% black garlic; and BG3: 3% black garlic; ns: not significant; LOD: Limit of detection; **: *p* < 0.01; ^a–c^: Means marked with different letters in the same column are statistically different (*p* < 0.05).

## Data Availability

The original contributions presented in the study are included in the article/Supplementary Material, and further inquiries can be directed to the corresponding author.

## Supplementary Materials

The following supporting information can be downloaded at https://www.mdpi.com/article/10.3390/foods14234055/s1; Figure S1: The calibration curve and chromatogram for nitrite analysis; Figure S2: The calibration curves and chromatograms for nitrosamine analysis; Table S1: The linear range, relative recoveries with RSDs, R2, LOD, LOQ, intra-day and inter-day precisions of N-nitrosamines.
