# Supplementary material for: Effects of Black Garlic Addition and Cooking Duration on Nitrosamine Levels and Quality Attributes of Sucuk
Source: Foods. 2025 Nov 26;14(23):4055. doi: 10.3390/foods14234055 (PMC12692503; doi:10.3390/foods14234055)
Supplement: Supplementary file 1 [file foods-14-04055-s001.zip › foods-4006776-supplementary.pdf]

Figure S1. The calibration curve and chromatogram for nitrite analysis

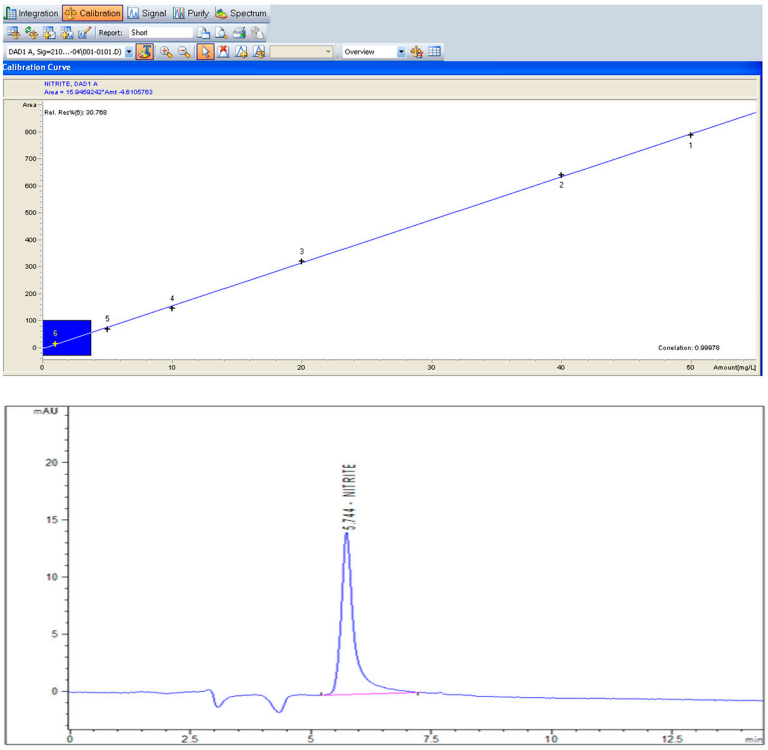

Table S1. The linear range, relative recoveries with RSDs, R2, LOD, LOQ, intra-day and inter-day precisions of N-nitrosamines

Nitrosamine	Linear range (µg L^{-1})	LOD	LOQ	R^2	Recovery (%)	RSD (%)	Inter-day RSD (%)	Intra-day RSD (%)
NDMA	0.5-20	0.32	0.98	0.9999	99.24-104.37	2.41-3.57	1.76	2.45
NMEA	0.5-20	0.42	1.27	0.9999	99.45-100.93	1.93-4.50	1.29	2.31
NDEA	0.5-20	0.44	1.34	0.9999	98.06-101.97	1.98-5.85	2.01	3.47
NDPA	0.5-20	0.42	1.36	0.9999	94.00-99.70	1.77-7.42	2.11	4.40
NPYR	0.5-20	0.36	1.09	0.9999	95.96-101.43	1.96-3.98	1.47	4.71
NPIP	0.5-20	0.15	0.46	0.9999	100.33-101.38	0.77-3.62	1.16	2.47
NDBA	0.5-20	0.38	1.15	0.9999	96.36-99.55	1.25-6.03	1.42	4.23

Figure S2. The calibration curves and chromatograms for nitrosamine analysis

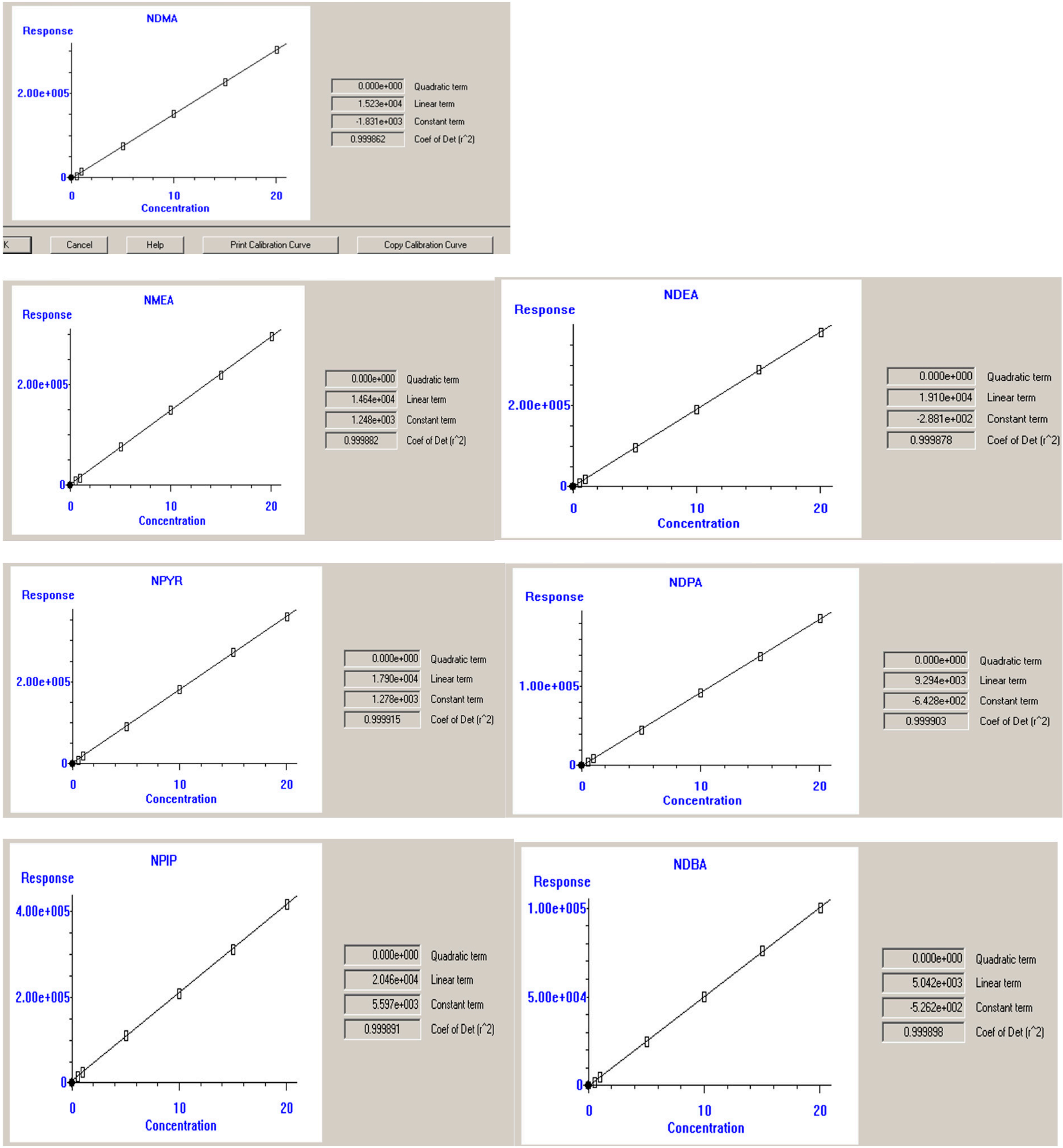

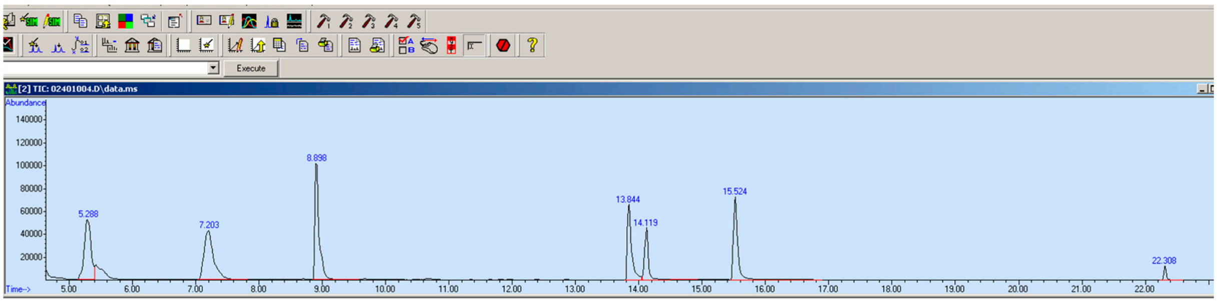
Execute
[2] TIC: 02401004.D\data.ms
Abundance
5.288
7.203
8.898
13.844
14.119
15.524
22.308
Time-->

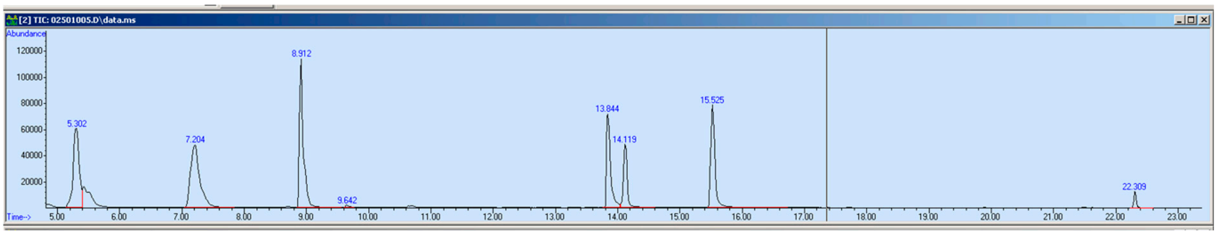
[2] TIC: 02501005.D\data.ms
Abundance
5.302
7.204
8.912
9.642
13.844
14.119
15.525
22.309
Time-->

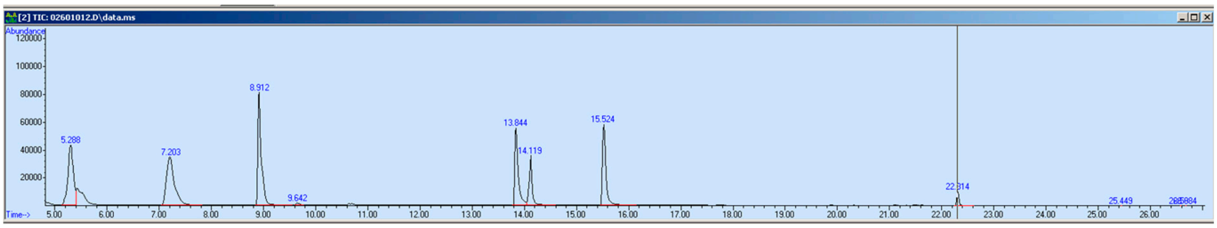
[2] TIC: 02601012.D\data.ms
Abundance
5.288
7.203
8.912
9.642
13.844
14.119
15.524
25.449
Time-->

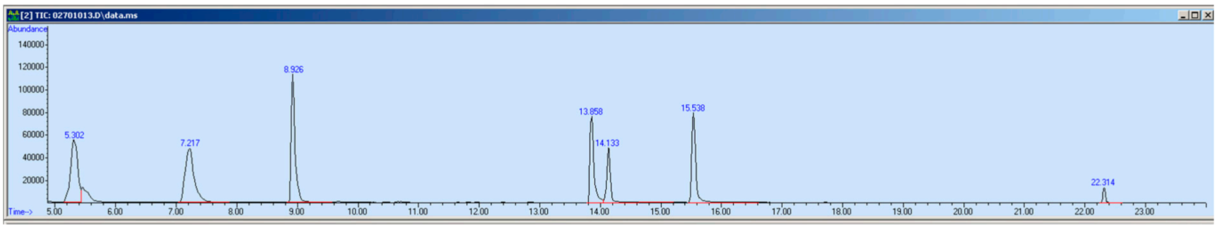
[2] TIC: 02701013.D\data.ms
Abundance
5.302
7.217
8.926
13.858
14.133
15.538
22.314
Time-->

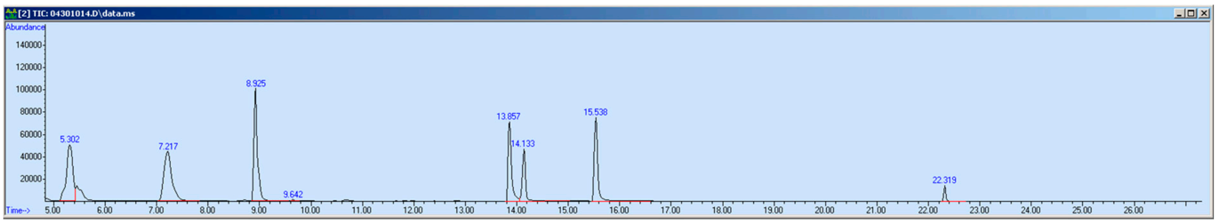
[2] TIC: 04301014.D\data.ms
Abundance
5.302
7.217
8.925
9.642
13.857
14.133
15.538
22.319
Time-->
